# Post-incident reviews—a gift to the Ward or just another procedure? Care providers’ experiences and considerations regarding post-incident reviews after restraint in mental health services. A qualitative study

**DOI:** 10.1186/s12913-020-05370-8

**Published:** 2020-06-03

**Authors:** Unn Elisabeth Hammervold, Reidun Norvoll, Kari Vevatne, Hildegunn Sagvaag

**Affiliations:** 1 Department of Public Health, Faculty of Health Sciences, University of Stavanger, 4036 Stavanger, Norway; 2 Work Research Institute, Oslo Metropolitan University, Oslo, Norway; 3 Department of care and ethics, Faculty of Health Sciences, University of Stavanger, Stavanger, Norway

**Keywords:** Post-incident reviews, Debriefing, Mental health, Restraint, Staff experiences, Participation, Care philosophy, Care ethics

## Abstract

**Abstract:**

Public guidelines in many western countries recommend post-incident reviews (PIRs) with patients after restraint use in mental health care. PIRs are one of several elements of seclusion and restraint reduction in internationally used programmes. PIRs may improve restraint prevention, patients’ recovery processes and care providers’ ethical mindfulness. The knowledge base on PIRs is, however, vague. This qualitative study explores professional care providers’ experiences and considerations regarding PIRs that included patients after restraint use in a Norwegian context.

**Methods:**

Within a phenomenological hermeneutical framework, 19 multidisciplinary care providers were interviewed about their experiences and views regarding PIRs that included patients after restraint events. The interviews were performed over the period 2015–2016. Data analysis followed a data-driven stepwise approach in line with thematic content analysis. A group of two patient consultants in mental health services, and one patient’s next of kin, contributed with input regarding the interview guide and analysis process.

**Results:**

Care providers experienced PIRs as having the potential to improve the quality of care through a) knowledge of other perspectives and solutions; b) increased ethical and professional awareness; and c) emotional and relational processing. However, the care providers considered that PIRs’ potential could be further exploited as they struggled to get hold on the patients’ voices in the encounter. The care providers considered that issue to be attributable to the patients’ conditions, the care providers’ safety and skills and the characteristics of institutional and cultural conditions.

**Conclusion:**

Human care philosophies and a framework of care ethics seem to be preconditions for promoting patients’ active participation in PIRs after restraints. Patients’ voices strengthen PIRs’ potential to improve care and may also contribute to restraint prevention. To minimise the power imbalance in PIRs, patients’ vulnerability, dependency and perceived dignity must be recognised. Patients’ individual needs and preferences should be assessed and mapped when planning PIRs, particularly regarding location, time and preferred participants. Care providers must receive training to strengthen their confidence in conducting PIRs in the best possible way. Patients’ experiences with PIRs should be explored, especially if participation by trusted family members, peers or advocates may support the patients in PIRs.

## Background

Post-incident reviews (PIRs) have been implemented in several western countries in recent years as part of seclusion and restraint (S/R) reduction programmes. Often referred to as the Six Core Strategies©, these programmes are underpinned by prevention- and trauma-informed principles. These programmes usually include a) leadership in organisational changes; b) the use of data to inform practice; c) workforce development; d) the use of S/R prevention tools; e) full inclusion of patients and their families; and f) rigorous debriefing that may include only care providers or both patients and care providers [1–3]. Restraint can be defined as a ‘mechanical or physical reaction against the patient including (the) use of straps, belts, other equipment or physically holding the patient preventing behaviour that might harm patients, care providers or (the) environment’ [4]. We rely on this definition in this research.

Mechanical restraint is widely considered to be among the most intrusive coercive measures, so the practice is controversial and contested [5, 6]. Consequently, much attention has been focused on restraint reduction in mental health services in recent decades [2, 7]. The international development of laws applicable to persons with psychosocial disabilities has encouraged more critical attitudes towards coercive measures, especially their use with people in vulnerable situations. This has prompted bans on all kinds of coercive measures [8]. In addition, ethical and professional imperatives urge developing reflexive practices aimed at limiting the use of coercion in morally justified cases and helping patients maintain hope and identity during crises, including the use of restraint [9, 10]. Despite the promise of these S/R reduction programmes, most studies on them have been based on development work aimed at S/R reduction, not rigorous research, so it is difficult to assess how much the different interventions have individually contributed to these supposedly promising results [2, 7, 11].

A PIR intervention is defined as ‘a complex intervention, taking place after a S/R episode and targeting the patient and healthcare team to enhance the care experience and provide meaningful learning for the patient, staff, and organisation’ ( [12],p.127). PIRs have recently been mandated by guidelines and laws in several countries, including Norway, even though their knowledge base is vague and does not require descriptions of the services’ value [2, 4]. The few studies conducted indicate that PIRs are usually implemented in services with defined care philosophies that are recovery based, strength based, person centred and trauma informed [11, 13]. These care philosophies are all founded on human values that emphasise a supportive environment, recognition of individual needs in care, and user participation by patients and care providers, both of whom are viewed as experts [9, 14, 15]. The literature describes PIR procedures including their timing, participants and themes. However, the broader question of how PIRs relate to the wider context of the organisation and culture is poorly described, although non-punitive, supportive approaches are recommended [16–18]. Human care philosophies with supportive approaches for conducting PIRs may conflict with the traditional organisation and culture of psychiatric institutions, often historically characterised by bureaucratic, hierarchical structures with paternalistic cultures that include habitual coercive practices and allow patients’ voices to have only marginal effects on services [10, 19].

In previous research, patients and care providers described PIRs as an arena for knowledge development, ethical reflection and recovery promotion [11]. Care providers also saw PIRs as beneficial as they increase professional reflexivity, which, in turn, results in improved care [11, 13, 20]. Furthermore, data from PIRs have been found to be useful for understanding patients’ experiences before, during and after restraint events; PIRs, therefore, are recommended to inform care plans [21]. These studies, however, included few participants, and their design and quality varied, so they have a low degree of comparability. In addition, they were published from 2001 to 2017, allowing time for different approaches concerning care philosophies in mental health services, as well as different contexts and issues, to influence how PIRs are conducted in practice [11].

Given PIRs’ potential in S/R reduction programmes, and the lack of knowledge about the core strategy of PIRs as a specific intervention and how they unfold in practice within mental health services, this research explores multidisciplinary care providers’ practical experiences and considerations concerning the use of PIRs after mechanical and physical restraint in mental health services in a Norwegian context. This multidisciplinary perspective is relevant as several kinds of professionals take part in PIRs. As this study is a part of a larger project, patients’ experiences and views on PIRs will be presented in another publication.

This research focuses on PIRs after physical and mechanical restraint events as these measures can have grave consequences, infringing upon patients’ human rights and risking physical and mental damage to both patients and care providers [22–25]. We therefore ask:

1. What are professional care providers’ experiences and considerations regarding the use of PIRs in practice?
2. What do professional care providers see as the benefits and challenges of PIRs?

The implications of the findings are discussed in relation to care ethics [26, 27] and a humanising care approach [28].

## Methods

### Design

To investigate professional care providers’ experiences with PIRs after the use of restraint in mental health services, we considered an explorative descriptive study design with a phenomenological hermeneutical approach, as this provided relevance to the study. To access care providers’ experiences and views concerning PIRs, we conducted qualitative interviews [29]. We found that Graneheim and Lundemann’s [30, 31] qualitative content analysis method was well suited to analyse multifaceted, sensitive, important phenomena regarding care, especially for a topic with such limited knowledge [32]. This analysis focused on the subject and context and offered opportunities to examine manifest and descriptive content and latent and interpretive content [30, 31]. In our study, the subject was the interviewees, the context was the mental health services wards, and the content was the care providers’ stories.

### Setting

We conducted the study in five locked wards in two mental health services in the same health region: a university hospital and a community mental health centre that both served patients with serious mental problems. These included psychoses or affective (bipolar) disorders, often also combined with addiction problems. The services had implemented PIRs as an intervention aimed at reducing the use of restraint but not as part of restraint reduction programmes. At the University hospital, they started an implementation project that included care providers from different ward units and a course for the employees, as well as monitoring PIR incidents. In the community mental health centre, the procedure was implemented somewhat differently over time in units were coercion was used. The two services had both written, formal procedures that were available in the start of the project. The services’ procedures were mostly congruent, but with some variation. The PIR procedures included questions about the patients’ and care providers’ comprehension of antecedents and potential triggers for restraint events, as well as the patients’ experiences of such events and their suggestions for alternative measures if similar occasions should arise. In addition, the care providers were asked about the basis for their decisions to use restraint and if they could have handled the situations differently. The patients were not asked the last question.

The PIR procedures varied according to time and participants. In the university hospital, PIRs had to be conducted within the procedural limit of 72 h from the restraint event, while in the community mental health centre, PIRs had to be conducted as soon as possible after the restraint event and no later than discharge. In that same service, the PIR participants included the patient, a milieu therapist who knew the patient, a responsible doctor or psychologist, the person responsible for the restraint decision and a relative based on the patient’s preferences. The university hospital’s procedures stated that PIRs should be conducted by a person not involved in the restraint incident and a care provider involved in the restraint event. This procedure also included an interpreter for non-Norwegian-speaking patients.

### Sample and recruitment

The participants were purposively recruited from the interdisciplinary population of the care providers at the two participating mental health services. The inclusion criteria were that the care providers had experience with PIRs that included patients after restraint use. The ward leaders and available care providers were given both written and oral information about the study. The care providers who gave consent were then contacted by the UH to set appointments for the interviews. Nineteen care providers agreed to participate, as shown in Table 1. First author, a mental health nurse with long-lasting experience from working in mental health services, was introduced to the participants as a ‘PhD-student’. Nobody withdrew their consent to participate in the study. The participants’ age ranged from 23 to 59 years, and their professional experience in mental health services ranged from 6 months to 25 years. Most providers had participated in PIRs less than five times, while four had participated more than five times.

**Table 1:**
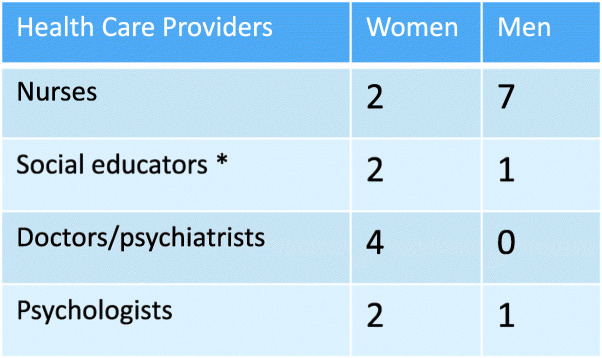
Overview of interdisciplinary participants

*Social educators included both health and social workers qualified to work in diverse, specialised health services

### Planning and conducting the interviews

Qualitative one-to-one interviews following a semi-structured interview guide were considered to be relevant to the exploration of the care providers’ views and experiences [29]. The guide included initial questions about the participants’ profession, age, years working in mental health services and experience conducting restraint and PIRs. The care providers were asked to briefly tell about a restraint event and then give a deeper description of how they experienced the PIR and share their thoughts about the patient’s experiences. During the development of the interview guide, a draft was presented to two patient consultants, experts with personal experience in mental health services [33, 34]. They gave valuable input that contributed to nuancing the UH’s preconceptions and so expanded the focus of the interviews. For example, the consultants viewed the definition of participants in PIRs as problematic.

The interviews were conducted in 2015 and 2016 in the participants’ ward units and lasted 17–51 min, with a mean time of 33 min. The interview guide was used to ensure that the most important issues were the themes in the dialogue, but room was allowed for other issues. The participants were asked to clarify when the interviewer did not understand their statements. After 19 interviews, we considered the information power as high, based on the criteria in the model of Malterud et al. [35], and we decided not to conduct more interviews. The interviews were tape-recorded and transcribed in verbatim in Norwegian.

### Analysis

The data analysis went ahead as follows. First, UH read through the interviews several times to obtain a sense of the whole. She also wrote notes on her immediate impressions of the interviews and ideas for the eventual theoretical framework [36, 37]. Second, she systematically identified the meaning units in the text, which were words, sentences and paragraphs whose content and context had related aspects. Two authors (UH and HS) condensed and labelled the meaning units into subcategories relevant to the study aim. NVivo 12 [38] was used as a tool in the analysis process.

Third, two authors (UH and HS) created categories answering ‘what’ questions related to the research questions and describing the manifest content of the text. The tentative categories were discussed by three researches (UH, HS and KV) and revised. To understand the participants’ experiences regarding PIRs’, the final step of the analysis was to formulate the latent content of the themes in collaboration with the co-authors. The emerging themes were placed against the transcribed text and the notes on first impressions in a hermeneutic circle and then recontextualized to achieve an overall understanding [30, 31]. Table 2 gives an example of qualitative content analyses, indicating the abstraction process from categories to theme.

**Table 2:**
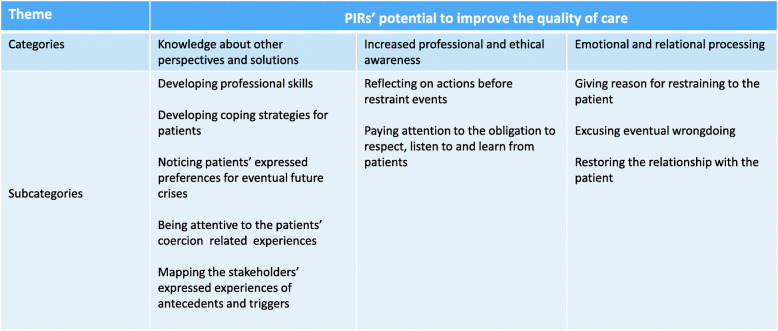
Theme, categories and subcategories

In the analysis process, reflexivity was emphasised through the exchange of ideas by the co-authors, and the project’s advisory group consisted of the two patient consultants in mental health services and the mother who was the next of kin to a patient. UH and the advisory group discussed preliminary results based on the care providers’ expressions in a two-hour meeting that was tape-recorded and listened to by UH as a supplement for reflections afterward. The discussions contributed other nuances and new questions that challenged the researchers’ preconceptions and consequently the preliminary results [37, 39, 40]. For example, the reference group attached importance to the patients’ vulnerability and power imbalance. Trustworthiness was considered to be important throughout the process and was strengthened by presenting the participants’ perspectives as faithfully as possible. A professional translator translated the quotations to ensure that the original interview text was maintained.

### Ethical considerations

The Norwegian Social Science Data Service (ref. no. 39122) assessed and approved the study, which followed ethical principles for research [41]. The Regional Committees on Health Research Ethics for Western Norway did not allow the researcher to be an observer in the ward units but decided that the study required no other ethical approval (2013/2359/REK south-east). In line with the Helsinki Declaration, the participants’ consent and confidentiality were secured, and they were provided with written and oral information including their right to withdraw at any stage without explanation or consequences [41].

## Results

The main results show a tension between care providers experiencing PIRs’ (1) *potential to improve the quality of care* and the experience of (2) *struggling to get hold of the patients’ voices* in the encounter. As the care providers struggled to get hold of the patients voices during the PIRs, PIRs’ potential did not seem to be utilised fully, which contradicted the aim of increasing dialogue between the care providers and patients.

### Potential to improve the quality of care

The care providers related PIRs’ potential to improve the quality care based on knowledge about other perspectives and solutions, as well as increase professional and ethical awareness and the care providers’ emotional and relational processing.

### Knowledge about other perspectives and solutions

The care providers described PIRs’ as beneficial due to their potential to develop new knowledge, mainly on the individual rather than the organisational level. They considered the new knowledge to be useful to prevent and minimise restraint events and to reduce harm when restraint seemed inevitable. The patients’ triggers before agitation were identified, such as excitement among the patients in the ward and the patients’ experiences of disrespect from the care providers. For example, a nurse quoted a former patient: *‘When you don’t meet me with respect, I get angry’* [7]. The nurse perceived that this was largely related to the way they spoke to the patient.

Informed of the patients’ expressed experiences, the care providers said that they developed insights into how their attitudes and behaviour could negatively influence the patients and consequently provoke situations in which the patients acted out. From the identification of these antecedents and triggers, alternative behaviours by the patients and care providers emerged. For example, when the patients became anxious, they could call on support from the care providers earlier. One patient suggested that a care provider ‘could snap one’s fingers in front of the patient’s eyes’ when he ‘was falling out’, an action the care provider [16] declared that she would not have thought of by herself.

Diversionary activities that stimulated interactions between the stakeholders emerged as possible restraint prevention measures, as in the following statement by a social educator:

*When we saw that her gaze started flickering, and she pulled away, we thought we might help to pull her back by focusing on something.* […] *Many of us had a liking for tea, so we’d bring different sorts of good teas, and then we’d sit trying to identify the scent. Was it fennel? Yes, maybe it was fennel. Or could it be cardamom? In this way, we managed to break the pattern that earlier would have developed into restraint situations or relocation to a shielded room* [16].

In accordance with the purpose of PIRs, the patients could share their restraint experiences during PIRs, and the stakeholders could work out how to conduct restraint with less strain on the patient if similar situations arose. As discussed by a nurse:

*We noticed that when we were lying on top of her, holding her, she cried out ‘No, Dad’. And afterwards, during our talk, it turned out she’d re-traumatised situations where her father had tied her up in bed and then abused her. It was the same setting* [11].

The nurse said that the information they received in the PIR initiated them to be more attentive to patients’ earlier experiences with sexual assault, and that they found mechanical restraint to be less strain for the woman.

Altogether, the care providers experienced PIRs as contributing valuable insights into restraint prevention and handling.

Although PIRs may have been an important knowledge source on restraint prevention, the care providers also experienced professional and ethical dilemmas regarding to what extent they could directly follow the patients’ suggestions within the framework of professional responsibility. One nurse quoted a former patient who they considered to be suicidal:

*You know, I’ve been told that ‘You should simply have let me go’. When we’ve had suicidal patients, who wish to harm themselves and to escape, they tend to come up with a solution that I can’t accept. That is because in my mind, it would not have been good for the patient* [15].

The patients’ suggestions that were considered to be related to their mental health, psychoses and suicidality demonstrated that the knowledge that emerged in PIRs could not be the only basis for actions. There was also a need for professional and ethical judgment.

### Increased professional and ethical awareness

The interviewees presented PIRs as an arena for reflection on restraint events, which stimulated their professional and ethical awareness and reflexivity concerning restraint use. Restraint events raised professional and ethical challenges in which different, potentially opposing values were at stake. Consequently, restraint events affected not only the patients but also the care providers. PIRs were, therefore, presented as a genuine opportunity for the persons involved to dwell upon the events together. As described by one nurse:

*So that you don’t just hurry on in a way that turns it into a forgotten incident. For such, such restraint incidents—to put it like that—using mechanical restraints, they’re obviously pretty strong experiences for the one who’s exposed to them but also for those who’re involved in them, aren’t they? They’re life events for, for the patient in a way and for the staff when it comes to that* [12].

The care providers viewed PIRs as stimulating both retrospective and prospective reflections. The retrospective reflections considered the past restraint event and the care providers’ arguments for the event and their handling of it. The certainty that restraint violated the patients’ human rights prompted the care providers to explore alternative measures. As one nurse said:

*But there’s something about taking such a situation seriously so that it does not become an abuse of power on our side, but that it is or that it was an act that was necessary there and then. And then one can always talk over afterwards what might have been done differently and why. However, I would say that on a more general basis, we’re becoming more conscious about our job, that’s to say the way we appear, so, yes, in general, there’s more ethical consciousness in our work* [14].

The care providers prospectively reflected on how to apply their new knowledge if similar occasions arose in the future. The care providers considered PIRs to have the potential to contribute to more individualised professional care by being an arena to hear the patients’ voices and preferences. Such care could decrease restraint events not morally or clinically justified.

### Emotional and relational processing

The care providers viewed PIRs as an arena to restore their damaged relationships with the patients. Generally, the care providers described mental strain related to conduct restraint and acknowledged that their handling of restraint could infringe upon the patients’ rights. Furthermore, they described a need to ‘*clear the air*’ (participant 15) by giving the patients explanations and, in some cases, excuses and justifications for the care providers’ restraint handling. In addition, contrasts between restraint use and everyday life in the unit emerged as an issue, especially for nurses and social educators, according to those working closest to the patients. One social educator [14] referred to what she experienced as a ‘*peculiar and demanding situation*’. One day, she restrained a patient, and the next morning, she entered that patient’s room to offer a cup of coffee. She argued that taking part in PIRs could decrease her discomfort in such situations.

The care providers stressed PIRs as beneficial for their processing of restraint events. As one doctor stated:

*It may be that health personnel feel it’s a major intervention, eh, to insert a needle or something like that,* […] *so it was important to say how I assessed the situation and where my perspective or experience met her* [the patient’s] *perception of reality in a way. In any case, afterwards, I felt it was good for my part* [1].

The care providers devoted less discussion to the benefits of PIRs for the patients’ processing but also pointed to opportunities for the patients to talk about their feelings of shame and guilt after acting-out episodes. Some care providers also mentioned the possibility of resuming PIRs later if the patients found something unclear or required elaboration concerning the issues discussed”. As stated by one nurse:

*Basically, I think it also would be an advantage if she* [the patient] *had any questions for me afterwards or if it was assumed that we had talked, being able to draw on the talk we had, the debriefing, that is* [12].

### Struggling to get hold of the patients’ voices

Half of the care providers expressed that they had experienced a failure to get hold of the patients’ voices in PIRs. They told that the patients tended to be passive and taciturn, as one nurse expressed; *Perhaps I thought the patient would be more verbal* [12]*.*

The care providers found that they experienced weak voices as challenging, since the PIRs did not contribute to alternative strategies for restraint prevention and handling.

For example, a doctor said:

*The patient was not very responsive. Almost no eye contact, so it was not possible to discuss the situation that had triggered the physical intervention etc., it was difficult to achieve it* [17]*.*

A psychologist described a similar experience:

*When we brought up what might have been done to avoid it* (restraint)*, it did not result in much conversation around it. It was more me asking her, and her not responding* [18]*.*

Regarding the care providers’ impression of the patients as passive, one nurse told that when asking the patient, she spoke with monosyllables. The care provider tried therefore to vary between open and closed questions

*It was mostly the moderator who asked, and the answers were typically monosyllables. However, we consistently invited her to talk, by asking open-ended questions, and closed questions* [11]*.*

The care providers presented different comprehensions about how to understand their struggle/failure to elicit the patients’ voices to deal with *patient related conditions, care provider related conditions* and *structural and cultural conditions.* The conditions are partly imbricated but will be presented separately.

### Patient related conditions

One reason given to help explain what the care providers perceived as passivity was the patients’ mental state when they took part in the PIR. Regarding the stated timeframe for PIRs, per PIR procedure, some care providers stressed the need to conduct the PIR within 72 h after restraint, but this resulted in some of the patients not having time to congregate after the restraint event.

One nurse said:

*But he is incapable of explaining things, so I believe the utility value of talking with him is not very great, at least the way he is now. He remembers very little of what happens, at least at present* [2]*.*

Consequently, the necessity to assess the patients’ health conditions before conducting the PIR was emphasised. As one nurse said:

*There’s not much purpose in having it* (a PIR) *when the patient is lying in a room placed in mechanical restraints or in seclusion compared to when the patient is sitting in a chair, experiencing oneself as independent—being seen and heard* [3].

Several care providers lifted patients’ difficult feelings as a possible explanation for the experienced passivity. One nurse [11] said that he perceived the patient as embarrassed in the PIR based on lot of hubbub when he was carried through the ward unit before he was restrained.

In another example a social educator expressed: *“I also believe quite many (patients) feel some guilt and shame afterwards”* [16]*.*

The care providers considered the patients’ conditions in PIRs to be characterized by confusion, shame, guilt and embarrassment. It was therefore challenging to meet the expectation of equivalence in the PIRs, and the HCP found themselves struggling to elicit the patients’ voices.

### Care provider related conditions

The care providers considered that their struggle to get hold of patients’ voices could be a result of their lack of skills regarding how to conduct the encounter in an optimal way and insecurity about their personal safety.

Some care providers reported that they received a minimum of information and training before their first PIR, which they assumed influenced their communication with the patients. A psychologist [19] described a clumsy approach when she conducted one PIR. She related that she, the nurse and the patient had different understandings of the restraint event and thus struggled to handle the situation. Consequently, PIRs became an arena for ‘fighting about the truth’. Further, moral uncertainty regarding PIRs surfaced, where it was seen as a mandatory procedure based on the risk ‘*to rip open old wounds after the situation had been calmed down*’ (doctor, 17).

Finally, PIRs as an arena for potential exposure to violence from the patients was presented and thus so was insecurity about the care providers’ personal safety in the PIR. As one nurse expressed: ‘*It’s a bad starting point for a good conversation when the staff are afraid of the patient*’ [3].

Therefore, PIRs sometimes included numerous care providers, which resulted in preponderance from the service in the encounter, an issue that was mentioned as one explanation for the patients’ passivity.

To increase their security, the care providers proposed professional reflection, information, education and ‘volume training’ in conducting PIRs.

### Structural and cultural conditions

The care providers presented several aspects that dealt with structural and cultural conditions that could affect the patients’ participation in the PIRs.

The arrangement of PIRs as ‘meetings’ which the patients were expected and told to attend was mentioned as a possible limiting factor for the patients. One social educator indicated that this approach could reinforce the patients’ sense of insecurity:

*And maybe it doesn’t suit the patient to have the conversation at that particular moment. It has something to do with—I’ve talked to a number of patients who say that ‘I always become nervous when I have to walk into some room or other to have a conversation’. You raise your shoulders, and you feel a bit on guard, yes. And that’s not really the best basis for recognising, sharing and communicating* (what you feel) [16]

She proposed alternatives, such as conducting PIRs by ‘*walk and talk*’ or ‘*sitting together in peace and quiet with a puzzle while talking*’. These alternatives addressed the patients’ discomfort regarding forms, checklists and the numerical imbalance putting the patients in a minority position relative to the care providers.

Another assumed reason for the patients to be taciturn in PIRs was overly rigid descriptions of who was to take part in them. The procedures seemed to be followed strictly, with the patient, doctor or psychologist and at least one nurse participating. This problem was compounded given the brief timeframe to conduct the PIR. Some care providers thought that PIRs could have been even more beneficial to the patients if conducted after a couple of days, allowing the participating care providers to build relationships of trust with the patients and increase the patients’ confidence in PIRs.

The care providers explained that the form of PIRs could hinder dialogue due to the patients’ previous experiences with a sometimes-overwhelming number of forms mapping them during their stays in services. The patients’ experiences of not being heard on other issues during their stay in services were another possible explanation for their passivity. The care providers, therefore, suggested also implementing PIRs after involuntary admissions, seclusion and forced medication to increase the patients’ confidence in PIRs as an arena for an honest exchange of views followed by changes in care plans.

Some interviewees described PIRs as an arena for confrontation and bringing up the patients, which could influence their participation. PIRs seemed to thus be marked by an approach focused on institutional rules, the patients’ deviant behaviour and a belief in the need for only the patients to learn lessons. The care providers’ perspectives on the antecedents of the restraint events were emphasised, and the patients’ deficits were stressed rather than their experiences and resources. These confrontations with the patients are illustrated by the nurse in the following example:

*Then we had a debriefing around the use of restraints and a debriefing around threats. It was the therapist, the patient, the care provider on that day and those who’d been involved in the incident. Those who’d been involved in the incident were called in one by one to report on how they had perceived the situation and the background to their views* [7].

Upbringing was further expressed in the care providers’ appeals to the patients’ common sense and responsibility, pointing to the patients’ previous utterances and behaviour. One nurse exemplified this approach: *“Sure, you may bring up that story* [the most recent restraint episode] *when ‘Yes, what happened then? What was it you said? What did you sort of promise me?”* [3].

The care providers acknowledged that basing PIRs on a form could negatively affect the patients but claimed that the form made them more confident in leading PIRs and further ensured that the right issues were discussed.

In practice, however, some interviewees experienced a need to seek individual approaches due to the patients’ health conditions and challenges stemming from the care providers’ shifts and the patients’ individual preferences.

Finally, the care providers reflected on how to utilise PIRs’ potential benefits by making their role in PIRs even more flexible. As one nurse expressed:

*It would have been a good thing, I believe, if we could have shifted focus a bit from what we as professionals have a duty to do, more to the effect of it, listen even more to the patient. And we should be humbler when it comes to whether we could do it in a different way* [12].

## Discussion

This study shows that care providers’ experience and considerations about PIRs’ have both possibilities and challenges, as PIRs’ potential to improve the quality of care may not be fully utilized because the care providers’ struggle to get hold of the patients’ voices. As responsiveness from the patients is a central moment in the caring process, the significance of getting hold to the patients’ voices in PIRs’ is a critical issue [26]. This condition is especially true for nurses and social educators who play key roles in the circumstances leading up to restraint events, conducting restraint events and taking care of the patients afterwards. In addition, care providers have an obligation, based on the moral and democratic imperatives, to include the perspectives of all those involved in the health care [27]. In order to understand the tension between PIRs’ potential to improve care and the challenge to get hold of the patients’ voices, we will focus on the care providers’ different approaches that will have an impact on the patients’ given roles in the PIRs [42]. We will first discuss the results where PIRs appear beneficial, then how staff struggled to bring out patients’ voices. Finally, we will discuss how the tension in results may be reduced in practice [26, 28].

### “A Gift to the Ward and Worth Its Weight in Gold When It Helps”

The results in this study indicate that care providers experience the PIR procedure as partly fulfilling the intentions of its being a tool to prevent the use of restraint use in mental health services; this is congruent with previous studies [11, 43]. The patients’ and care providers’ mutual reflections in PIRs may give an overview of antecedents, restraint implementing and patients’ experiences and considerations regarding alternative measures in the aftermath. This *reflection on action* [44] highlights alternative, more person-centred solutions should a new crisis occur, and is based on the patients’ personal knowledge that is the epistemological base of recovery oriented practices [9]. Consequently, the care providers can provide improved care to patients in a crisis based on strengthened professional knowledge and increased awareness of the moral elements of care such as attentiveness, responsibility, and responsiveness [11, 26, 45].

Originally, PIRs were introduced in S/R prevention programmes grounded in human-based care philosophies, including full inclusion of patients and their families [1]. That means changed roles for both patients and care providers [9, 42]. To the patient, that implies a changed role from passive receiver of care to active agent. In the frame of personal recovery, a crisis is an active space which can contribute to growth [9, 46]. The care providers’ role will then be to minimise the loss of the patients’ responsibility such as asking the patient in the PIR if one could have handled what happened before the restraint event in another way so that the result could have been less thorough. Further, the care providers’ task is to communicate and represent hope during crises and support the patients’ identity during and after the event [9]. Participation in PIRs may with that promote the patients’ personal recovery processes by supporting empowerment, hope and identity [9, 13, 18, 20, 21, 47, 48].

Previous studies emphasise the necessity of supportive environments in PIRs [13, 16, 18, 49]. The care providers that experienced PIRs as beneficial seemed to perform an acknowledging, dialogue-oriented approach with the patients. This approach is characterised by the values of care ethics and a humanising framework that may support the patients’ human dimensions and thereby support empowerment processes and strengthen their voices in PIRs [28, 50].

Care providers’ experiences and views on PIRs’ potential for emotional and relational processing are in line with previous studies that suggest PIRs help the care providers’ deal with emotional and moral stress [10, 11, 13, 49]. As PIRs with the patient mainly focus on the patients’ experiences and considerations, care providers’ personal needs for defusing after restraint events belongs to other arenas [1, 11, 51]. From patients’ perspectives, we have in our review [11] found one small study suggesting PIRs are beneficial regarding patients’ processing of the restraint incident [47]. Based on the scarce prevailing literature and the patients’ vulnerable and dependent role in the services, we cannot conclude that the potential to process is transferable to the patients.

Based on their role as inpatients and actual health condition, the patients are for some time dependent on the care providers who have professional knowledge and the ability to help, and with that also power. Consequently, a power imbalance exists in the patient–care provider relationship, something that becomes further enhanced by restraint use [26, 52–55]. According to Emerson [56], power (in this case, the care providers’ power) resides implicitly in the dependence of others (in this case, the patients). To achieve more balance in power–dependence relationships, the weaker members’ power should be increased. Aiming to give the patients increased status in PIRs, the care providers should increase their motivational investment in the goals defined by the patients [56]. The patients’ personal goals will be individual, but based on previous studies they may deal with the services’ relational, structural and cultural conditions [23, 55, 57, 58]. An assumption for identifying the patients’ goals in the PIRs is a supportive environment in an ‘I–thou relationship’ ( [59]) where the care providers acknowledge the participants’ roles to be approximate equal. Equal roles in the relationships in health services are, however, impossible in practice based on the power imbalance. A frame of care ethics may nevertheless support the patients’ participation in PIRs based on the recognition of the context, the power imbalance and the moral claim to include everyone’s perspectives in care [26, 27].

### Perhaps I thought the patient would be more verbal

We have not found previous studies that describe the challenge to elicit patients’ voices in the PIRs. Our results however indicate that some care providers’ approaches and statements may reinforce the power–dependence relationship by an authoritarian approach that emphasises the patients’ deviant behaviour and deficits rather than their experiences and resources [56, 60]. Further, an appeal by the care providers to the patients’ common sense and responsibility before the restraint event may result in PIRs becoming an arena for conflict where the patients are confronted with, − and made responsible for their previous behaviours and statements. In light of Foucault, we understand this authoritarian measure as *a disciplining approach* [61] that may mobilise the patients’ counter-power and counter-behaviour [62, 63]. Counter-behaviour can be active resistance due to a lack of other strategies available to those in dependent situations [e.g. inpatients] or protest reactions such as taking a passive role with withdrawal and attempts to evade contact [64]. The last point may be one of several explanations for the patients’ passivity in PIRs, as this disciplining approach may contribute to silencing voices [61]. A disciplining approach in PIRs seems to be characterised by an ‘I–it relationship’, a subject–object relationship in which, according to Buber, ‘the object exists only through being bounded by others’ (( [59]),p.12). As the patients are vulnerable and mortified during and after being restrained, they need to be met as unique subjects with care, respect and empathy [43, 55, 65]. To ask the patient in the PIR whether one could have handled the situation in another way may consequently be experienced as blaming and an additional burden [55]. Being met with a disciplining approach in the PIR, and subsequently detached care, may thus be experienced as a prolongation of coercive practices that confirm the identity as patient in a passive role and thus hamper ones’ personal recovery processes [19].

In order to achieve more balance in the power-dependence relationship and thus increase the patient’s power in PIRs, the patient’s preferences regarding point in time, participants and context for the encounter should be recognised. In previous studies, inclusion of family members or peers in PIRs is suggested [13, 21]. The interviewees in this study did not mention that possibility, even though the community mental health centre’s procedure suggested a next of kin as participant. Advocacy can also, by their role, provide a counterbalance, to ensure that the individual’s personal perspectives are represented and heard in the PIR, further supporting the patients’ empowerment processes [66, 67].

Finally, implementing PIRs in the two participating services was not based on defined care philosophies as described in several studies [16, 17, 48, 68]. Even though some care providers presented values and approaches in line with human based care philosophies and even struggled to get hold of the patients’ voices in the PIR, the services’ context seemed still to be characterized on traditional clinical recovery model’s values, goals and working practices [9, 10, 19]. Consequently, to implement PIR in line with the intentions, presupposes attention to the structural and cultural conditions in the services by recognising the power imbalance between patients and care providers’, and devotes attention to the necessity of a supportive and recognising context when conducting PIRs [11, 26, 60].

## Methodological considerations

A strength of this study is that we have investigated a fairly unexplored field of knowledge. In addition to contributing to the knowledge base, this study reveals a need for more exploration of PIRs. We also consider it to be a strength that three researchers participated in the analysis process, comparing the meaning units and discussing the subthemes and themes. The reference group’s input to the interview guide and preliminary results helped nuance the materials and strengthen user perspectives as experts by experience [33].

The study was conducted by interviewing the care providers who volunteered to part, suggesting a potential bias in the study population. The results are context specific, which means that they show the participating care providers’ experiences. The core results are, however, consistent with previous research on the utility value of PIRs [11, 12, 69]. Repeat interviews or feedback to the participants were not provided, which could further have strengthened the results. We only have data on the care providers’ experiences and considerations from particular interview situations. The regional ethical committee did not approve observations of the services, so we have no observations or information on the restraint episodes and the patients’ views that could have contributed to the context. The patients’ perspectives, therefore, could contribute other considerations and viewpoints that need to be further explored. None of the authors were connected to the two participating mental health services, which could have been a limitation.

## Conclusion

The main study findings are that care providers experience a tension between PIRs’ potential to improve the quality of care and their struggle to get hold of the patients’ voices. To motivate the patients’ active participation, PIRs should be conducted within the context of a humanising care approach. A framework of care ethics may guide the care providers to plan PIRs along with the patients’ preferences and further conduct PIRs’ in a collaborative, supportive atmosphere that promotes the patients ‘personal recovery processes. Studies exploring the patients’ experiences with PIRs are lacking, thus there is a need for further research to get hold of their perspectives. Of pivotal interest is PIRs’ potential for the patients’ emotional and relational processing and the possible utility value of support from family members, peers or advocacy in the encounters. To strengthen the care providers confidence and communication skills in the PIRs’ there seems to be a need for education, professional reflection opportunities and sufficient training.

## Data Availability

The transcripts from the interviews are confidential and will not be shared.

## Abbreviations

PIR: Post Incident Review
S/R: Seclusion and Restraint
